# Associations between neurocognitive functioning and social and occupational resilience among South African women exposed to childhood trauma

**DOI:** 10.1080/20008198.2017.1394146

**Published:** 2017-11-02

**Authors:** C. A. Denckla, N. S. Consedine, G. Spies, M. Cherner, D. C. Henderson, K. C. Koenen, S. Seedat

**Affiliations:** ^a^ Department of Epidemiology, Harvard T. H. Chan School of Public Health, Cambridge, MA, USA; ^b^ Department of Psychological Medicine, University of Auckland, Auckland, New Zealand; ^c^ Department of Psychiatry, Stellenbosch University, Cape Town, South Africa; ^d^ HIV Neurobehavioral Research Center, University of California, San Diego, CA, USA; ^e^ Boston Medical Center, Boston University, Boston, MA, USA

**Keywords:** Trauma, resilience, Sub-Saharan Africa, childhood abuse, trauma, resiliencia, Africa Sub-sahariana, abuso infantil, 创伤，韧性，撒哈拉以南非洲，童年创伤, • Memory and executive functioning have effects on posttraumatic exposure psychopathologies.• Women with lower non-verbal memory displayed greater social and occupational resilience.• Women with lower sematic language fluency and processing speed evidenced improved occupational resilience.

## Abstract

**Background**: Prior research on adaptation after early trauma among black South African women typically assessed resilience in ways that lacked contextual specificity. In addition, the neurocognitive correlates of social and occupational resilience have not been investigated.

**Objective**: The primary aim of this exploratory study was to identify domains of neurocognitive functioning associated with social and occupational resilience, defined as functioning at a level beyond what would be expected given exposure to childhood trauma.

**Methods**: A sample of black South African women, *N* = 314, completed a neuropsychological battery, a questionnaire assessing exposure to childhood trauma, and self-report measures of functional status. We generated indices of social and occupational resilience by regressing childhood trauma exposure on social and occupational functioning, saving the residuals as indices of social and occupational functioning beyond what would be expected given exposure to childhood trauma.

**Results**: Women with lower non-verbal memory evidenced greater social and occupational resilience above and beyond the effects attributable to age, education, HIV status, and depressive and posttraumatic stress symptoms. In addition, women with greater occupational resilience exhibited lower semantic language fluency and processing speed.

**Conclusion**: Results are somewhat consistent with prior studies implicating memory effects in impairment following trauma, though our findings suggest that *reduced* abilities in these domains may be associated with greater resilience. Studies that use prospective designs and objective assessment of functional status are needed to determine whether non-verbal memory, semantic fluency, and processing speed are implicated in the neural circuitry of post-traumatic exposure resilience.

## Abstract (es)

**Planteamiento**: Las investigaciones previas sobre la adaptación después del trauma temprano entre las mujeres negras de Sudáfrica generalmente evaluaban la resiliencia de maneras que carecían de especificidad contextual. Además, no se han investigados los correlatos neurocognitivos de la resiliencia social y ocupacional.

**Objetivo**: El objetivo principal de este estudio exploratorio fue identificar los dominios del funcionamiento neurocognitivo asociados con la resiliencia social y ocupacional, que se define como el funcionamiento en un nivel mayor de lo que se esperaría dada la exposición al trauma infantil.

**Métodos**: Una muestra de mujeres negras sudafricanas, *N* = 314, completó una batería neuropsicológica, un cuestionario que evaluaba la exposición al trauma infantil y medidas de auto informe de su funcionamiento. Generamos índices de resiliencia social y ocupacional mediante la regresión de la exposición al trauma infantil en el funcionamiento social y laboral, conservando los residuos como índices de funcionamiento social y laboral más allá de lo esperado dada la exposición al trauma infantil.

**Resultados**: Las mujeres con menor memoria no verbal mostraron una mayor resiliencia social y ocupacional por encima y más allá de los efectos atribuibles a la edad, la educación, el estado del VIH y los síntomas de depresión y estrés postraumático. Además, las mujeres con mayor resiliencia ocupacional mostraron menor fluidez del lenguaje semántico y de velocidad de procesamiento.

**Conclusión**: Los resultados son algo consistentes con los estudios previos que implican los efectos de la memoria en el deterioro después del trauma, aunque nuestros hallazgos sugieren que las habilidades reducidas en estos dominios pueden asociarse a una mayor resiliencia. Se necesitan estudios que usen diseños prospectivos y una evaluación objetiva del estado funcional para determinar si la memoria no verbal, la fluidez semántica y la velocidad de procesamiento están implicadas en los circuitos neuronales de la resiliencia a la exposición postraumática.

## Abstract (zh)

**背景:** 关于黑肤色南非女性早期创伤适应的前人研究总是用缺乏内容特异性的方式评估韧性。并且，社会和职业韧性的神经认知关联还没有被研究过。

**目标:** 这个探索性研究的目的是识别和社会与职业韧性（童年创伤后超过预期的功能水平）有关的神经认知功能。

**方法:** 样本包括314名黑肤色的南非女性，完成了一组神经心理学问卷：包括童年创伤暴露和功能状态的自评问卷。我们将童年创伤暴露回归在社会和职业功能上，保存残差作为经历童年创伤后超过预期水平的社会和职业功能指标，由此得到社会和职业韧性指标。

**结果:** 控制年龄、教育、HIV、抑郁和创伤后应激症状后，非言语记忆较低的女性表现出更高的社会和职业韧性。另外，较高职业韧性的女性表现出更低的语义语言流畅性和加工速度。

**结论:** 结果和前人研究一定程度上一致，说明记忆对创伤之后损伤的影响，尽管我们的发现提示在这些方面的能力降低可能和更高的韧性有关。还需要进行前瞻设计研究和对功能状态进行客观测量来决定非言语记忆、语义流畅度和加工速度是否和创伤暴露后韧性的神经环路有关。

South African women are exposed to high rates of adverse experiences in childhood including physical punishment (89.3%), physical hardship (65.8%), emotional abuse (54.7%), emotional neglect (41.6%), and sexual abuse (39.1%) (Jewkes, Dunkle, Nduna, Jama, & Puren, 2010). Despite such high rates of exposure, the lifetime prevalence of posttraumatic stress disorder estimated in a nationally representative study of South Africans was 2.3% (Atwoli et al., 2013). More is known about the negative health consequences of exposure to these adversities, including poorer physical and mental health decades after trauma (Springer, Sheridan, Kuo, & Carnes, 2007), than about the protective factors that might predict preserved functioning. Despite being overlooked, however, the fact that not everyone who is exposed develops ensuing psychopathology means it is possible that there are pathways which buffer the deleterious effects of exposures. This study focuses on candidate neurocognitive domains, aiming to identify pathways associated with social and occupational resilience among South African women exposed to childhood trauma, defined as physical, sexual, and emotional abuse, and physical and emotional neglect.

The range of operational and theoretical approaches to studying resilience make defining terms essential to interpreting results. Most contemporary scholars define resilience as preservation of functioning following exposure to acutely adverse experiences (Bonanno, 2004, 2012; Luthar, Cicchetti, & Becker, 2000). In the developmental context, Masten (2014, p. 6) defines resilience as, ‘the capacity of a dynamic system to adapt successfully to disturbances that threaten the viability, the function, or the development of that system.’ This systems model suggests that the capacity for adaptation is distributed across interacting systems. In the present report, we focus on the interacting systems of neurocognition, functional status, and culturally diverse contexts to investigate factors potentially influencing resilient functioning among adult women.

Although studies investigating the neurocognitive correlates of resilience are notably absent in the South African context, prior studies have sought to identify relevant psychosocial factors (Greeff & Loubser, 2008; Phasha, 2009; Spies & Seedat, 2014). One study found that trait resilience, assessed using the Connor-Davidson Resilience Scale (CD-RISC; Connor & Davidson, 2003), was associated with reduced depression among trauma-exposed, HIV-infected women (Spies & Seedat, 2014). Another study found that optimism, prosocial behaviour, and a future-orientation were associated with women’s educational attainment following exposure to severe sexual abuse (Phasha, 2009). Among isiXhosa-speaking families, spirituality has been associated with families’ ability to negotiate successful adaptation after a crisis (Greeff & Loubser, 2008). However, given the variability in approaches to measuring resilience in these studies, few general conclusions can be made about patterns of resilience in the South African context.

One initial problem confronting researchers examining resilience in samples of diverse traumatized women is the scarcity of measurement approaches that reflect specificity with respect to (a) the specific domains of functioning being examined or (b) sensitivity to the type of traumatic exposure. Self-report instruments of resilience do not incorporate measures of event exposure(s) or offer a specific index of preservation of functioning (see Denckla and Mancini, 2016, for details). To address these limitations, the present study employed an index of resilience used in prior studies (Consedine, Magai, & Conway, 2004; Consedine, Magai, & Krivoshekova, 2005; Hayman, Kerse, & Consedine, 2016) that estimates functioning in social and occupational domains beyond what would be expected given prior exposure to childhood trauma. Specifically, this index is generated by regressing childhood trauma exposure on social and occupational functioning and treating the standardized residuals as an index of resilience (see Methods for further details). This approach allowed us to highlight domains of adaptive functioning that are relevant to this population specifically, and to estimate functioning relative to others in the sample.

A related limitation in prior research is that many measures lack contextual sensitivity to the domains of functioning in which preservation actually matters. While aspects of personality predict responses to stressors (Block & Block, 1980), measurement approaches based only on *trait* predictors of adaptive outcome say little about the different challenges that diverse samples face or the specific domains of functioning in which resilience manifests (Bonanno, 2012; Hayman et al., 2016). In the current study, we examined a commonly occurring trauma among South African women and assessed outcomes of high contextual relevance to the sample. Contextual relevance was based on (1) prior qualitative studies in the target population suggesting that prosocial behaviour was associated with greater educational attainment following women’s exposure to severe sexual abuse (Phasha, 2009), and (2) reasoning that a measure of occupational resilience would be especially salient given the under-resourced economic and instrumental contexts in which these women carry out their daily lives. This general approach has been used in prior studies of diverse samples (Consedine et al., 2004) and is advantaged insofar as it ensures that measurement and operationalization are contextually relevant to both (a) the common traumas and (b) adaptive outcomes of importance to the sample (see Hayman et al., 2016, for a similar approach).

Finally, prior work in this area has directed little effort to identifying performance-based measurements of cognitive functioning associated with resilience. More research is needed because we know that different components of fear memory formulation and modulation may help explain why some people recover from heightened fear after exposure to traumatic events while others experience pathological sequelae or fail to recover (Jovanovic et al., 2006; Parsons & Ressler, 2013; Yehuda, Flory, Southwick, & Charney, 2006). Studies have found that both verbal and non-verbal learning are associated with posttraumatic stress disorder among combat exposed individuals (Jelinek et al., 2006; Scott et al., 2015; Vasterling et al., 2006). For example, among a highly exposed, racially diverse sample of urban dwelling adults, asymptomatic adults displayed better nonverbal memory compared to their symptomatic counterparts (Wingo, Fani, Bradley, & Ressler, 2010). Further insight into the specific elements of cognitive functioning that predict more versus less resilient responses to trauma exposure may suggest remediable factors that could inform intervention (see Gould et al., 2012; Teicher, Samson, Anderson, & Ohashi, 2016).

To summarize, the primary aim of this study was to identify the specific neurocognitive domains associated with social and occupational resilience among South African women with prior exposure to childhood abuse. To achieve this aim, this study integrated three methodological approaches that offered advantages over prior work in this area: (1) Consistent with definitions of resilience, we operationalized resilience in terms of functioning relative to traumatic exposure using a regression-based approach; (2) We employed outcome indices of resilience that accessed contextually relevant functioning, that is, functioning in domains of primary relevance to the daily context in which the women in our sample live; and (3) We used objective, performance-based neurocognitive measures to better understand basic cognitive pathways implicated in recovery from exposure to trauma rather than a self-report measure. Given that prior work on asymptomatic trauma exposed adults has found evidence for better non-verbal memory compared to their symptomatic counterparts (Wingo et al., 2010), we hypothesized that superior non-verbal memory would predict better functioning in social and occupational domains beyond what would be expected given prior exposure to childhood abuse.

## 1. Methods

### 1.1. Participants

Participants were enrolled in a study investigating the behavioural and brain effects of childhood abuse and HIV-infection in South African women. Women were eligible to participate in the broader study if they were between the ages of 18 and 65 years, able to give written consent, able to read and write in English, Afrikaans, or isiXhosa (an indigenous African language spoken natively in South Africa) at 5th grade level, had no history of psychotropic medications, and were medically well enough to undergo neuropsychological testing.

The sample examined in the present report included 314 women with a mean age of 30.7 years (range 18–50), was predominantly black (98.3%), and isiXhosa-speaking (94.9%). The majority were unemployed (71.6%) and single (70.3%), with an average number of years of education of 10.53 years (range 5–14 years). Approximately half of the participants were HIV positive (47.3%) (see Table 1).

### 1.2. Procedure

The ethics board of Stellenbosch University, Cape Town, South Africa, approved the study and participants provided written informed consent. Potentially eligible HIV-positive and HIV-negative women were recruited from hospitals/day clinics and communities around Cape Town. Participants were reimbursed ZAR250.00 (c. US$20) for transportation costs to the study site. A trained research psychologist and a research nurse administered the neuropsychological battery. Tests were administered in English, Afrikaans, or isiXhosa according to the language participants’ self-reported as being spoken in the home. Consistent with standard ethnographic practice, test instructions and stimuli were translated into Afrikaans and isiXhosa using standard test adaptation techniques including forward and back translations.

### 1.3. Measures

#### 1.3.1. Childhood trauma

The Childhood Trauma Questionnaire – Short Form (CTQ-SF; Bernstein et al., 2003) is a retrospective self-report screening measure for childhood abuse and neglect for both clinical and non-clinical populations. It contains 28 items that are rated on a 5-point scale ranging from 1 *= never true* to 5 = *very often true*. These 28 items comprise five clinical scales consisting of physical, sexual, and emotional abuse, and physical and emotional neglect. Sample items include ‘People in my family hit me so hard that it left bruises or marks’ and ‘I believe that I was sexually abused.’ Some items are reverse scored, such as ‘I had the best family in the world.’ After reverse scoring designated items, responses are summed to derive a total score with a range from 25 to 116. Reliability for the current sample was α = .83.

#### 1.3.2. Functional status

The Quality of Life Enjoyment and Satisfaction Questionnaire (Q-LES-Q; Endicott, Nee, Harrison, & Blumenthal, 1993) is a self-report measure assessing enjoyment and satisfaction across functioning in social (11 items), occupational (13 items), household (10 items), physical (13 items), emotional (14 items), school (10 items), leisure (6 items), and general (16 items) domains. Items are rated on a 5-point scale ranging from 1 = *never/not at all* to 5 = *frequently or all of the time*. Items on the two indices that assess social and household functioning were employed for subsequent analyses. We use the term occupational functioning in place of household functioning for two reasons: (1) Women in this sample report low rates of employment and there is insufficient data on the occupational functioning index of the Q-LES-Q; and (2) We reasoned that the ability to carry out household duties was an acceptable proxy assessment of functional occupational status. Sample items from the household functioning (henceforth occupational functioning) domain include ‘Prepared food or obtained food to your satisfaction?’ and ‘Kept your room/apartment/house cleaned to your satisfaction?’ Sample items from the social functioning domain include ‘Enjoyed talking with or being with friends or relatives?’ and ‘Felt your relationships with your friends or relatives were without major problems or conflicts?’ Responses are summed to derive a total score ranging from 11 to 55 for the social functioning scale and 10 to 50 for the household functioning scale, with increasing total scores representing greater functional status. Reliability for the current sample was α = .90 for social functioning and α = .85 for the household functioning subscales.

#### 1.3.3. Resilience

For the purposes of the current study, resilience was defined as functionality *relative* to childhood abuse (see Consedine et al., 2004, 2005; Hayman et al., 2016 for similar approaches). Consistent with prior studies, the resilience score considered in the present report was derived by regressing the total childhood trauma score on the total household functioning score (henceforth referred to as occupational resilience), *F* 1, 310 = 7.48, *p* = .007, *R^2^* = .024, and total social functioning scores (henceforth referred to as social resilience), *F* 1, 310 = 3.20, *p* = .075, *R^2^* = .01, treating the standardized residuals (observed – expected) as an index of resilience. The two indices were significantly correlated, *r* = .68, *p* = .000. In this approach, persons with residuals above the line of best fit represent those who are functioning better than what would be expected given their abuse exposure, while the reverse is true for those scoring below the line.

#### 1.3.4. Neurocognitive domains

We administered a standard neurocognitive battery assessing seven domains (learning, delayed recall, processing speed, attention/working memory, executive function, verbal fluency, and motor ability) typically administered in HIV research (Heaton et al., 2010). Neurocognitive tests were adapted to the local South African context (Spies, Fennema-Notestine, Cherner, & Seedat, 2017), with specific cultural modifications for the South African context made to the Hopkins Verbal Learning Test-Revised (HVLT-R) (replacing precious stones with vegetables) and the Controlled Oral Word Association Task (COWAT) (changing the letter stimulus).

#### 1.3.5. Psychiatric symptoms

The Center for Epidemiologic Studies Depression Scale (CES-D; Radolff, 1977) is a 20-item self-report screen for symptoms of current depression (i.e. experienced in the past week). The possible range of scores is 0 to 60, with higher total scores indicating more symptoms. The Davidson Trauma Scale (DTS; Davidson et al., 1997) is a 17-item, self-rating scale of PTSD symptoms corresponding to the DSM-IV (American Psychiatric Association, 2000) symptom criteria of PTSD. Higher scores indicate greater PTSD symptomology, with the total score computed by summing self-report ratings of both frequency and severity of each symptom item.

## 2. Analytic strategy

Our analysis proceeded in three phases. First, we characterized the demographic features of our sample including age, education, marital status, household income, ethnicity, and language spoken at home. We then conducted a series of univariate correlations to examine the associations between social and occupational functioning, depression and trauma symptoms, resilience, and neurocognitive domains. Next, we proceeded with a focused analysis of the significant associations evident in the univariate correlations by conducting a series of hierarchical multivariate regressions. As age, education, and HIV status are known to predict neurocognitive performance (Devlin et al., 2012), as well as depressive symptoms (McClintock, Husain, Greer, & Cullum, 2010) and Posttraumatic Stress Disorder (PTSD; American Psychiatric Association, 2000; Scott et al., 2015), these variables were entered in the first step to capture within-source variability. Then, neuropsychological variables were entered in the second step to measure between-source variability, thereby isolating the variance explained by our neuropsychological variables. Because validated norms for neuropsychological tests are not yet available for isiXhosa speaking South African women, we proceeded with an analysis using raw scores. Given that our sample was of a single gender with moderate variability in age and education, we reasoned that this approach was acceptable given the lack of existing norms. Finally, because we were ultimately interested in the differential associations between neuropsychological domains and resilience, we elected to specify separate hierarchical multiple regression models for each neurocognitive test because we reasoned that individual neuropsychological tests demonstrate sufficient independence from one another in terms of the target neuropsychological domain, as well as the method in which different tests are executed (e.g. some require only verbal response, others require written response, some are executed on a computer, etc.).

## 3. Results

We first characterized sample demographics as well as the clinical and trauma exposure characteristics of our sample (see Table 1). On average, women in the sample reported moderate levels of childhood abuse, *M* = 46.49, *SD* = 19.28. The mean value for household quality of life was 4.41 (.83), range 0–5, and the mean value for social quality of life was 4.24 (.84), range 0–5.

**Table 1.** Socio-demographic characteristics of participants, *N* = 314.

	Mean (*SD*) or %
Age	30.71 (7.91)
Ethnicity	
Black	98.3%
Coloured	1.7%
Home language	
English	1.4%
isiXhosa	94.9%
Other	3.7%
Education	
Grade 8 or less	10.8%
Grade 9–12	86.2%
Diploma	1.7%
University degree	1.3%
Household income	
Less than R10,000 (US$781)	86.8%
More than R10,000	13.2%
Primary breadwinner (yes)	32.4%
Marital status	
Married/cohabitating	25.7%
Separated/divorced/widowed	4.0%
Single	70.3%
HIV Status (+)	47.3%
Employed (yes)	28.4%
Number of children	1.57 (1.24)

Next, we examined zero order correlations between selected neurocognitive tests used in prior studies (Wingo et al., 2010), five domains of functioning as assessed by the Q-LES-Q for which we had representative data: physical health, feelings, household duties, hobbies, social relations, and general activities, depression and trauma symptoms, childhood abuse severity, and derived resilience measures. Means, standard deviations, and correlations are reported in Table 2. Contrary to expectations, functioning in social and household domains demonstrated *negative* significant correlations between neurocognitive tests in two domains: processing speed, and learning and recall. Associations among social and occupational resilience were significantly *negatively* associated with BVMT-R non-verbal learning and recall, HVLT-R recall, and WAIS-III digit-symbol. Verbal language category fluency (animals) and WAIS-III symbol search were also significantly *negatively* associated with occupational resilience.

**Table 2.** Means, standard deviations, and zero order correlations between raw neurocognitive scores and Quality of Life Enjoyment and Satisfaction Questionnaire (Q-LES-Q) domains of functioning, depressive and trauma symptoms, and childhood abuse severity.

Neurocognitive Domain	Neurocognitive Test	Mean (*SD*)	Physical Health	Feelings	Household	Hobbies	Social	General Activities	Social Resil.	Occupation Resil.
Processing Speed	WAIS-III DS	47.60 (15.06)	.03	.05	−.13*	.01	−.11	.01	−.15*	−.18**
	WAIS-III SS	17.36 (8.34)	.02	.04	−.13*	.04	−.09	.02	−.11	−.16**
Attention/Working	PASAT	22.68 (9.63)	.02	.03	−.08	.01	−.06	.03	−.08	−.10
Memory	WMS-III SS	11.35 (3.30)	.02	.05	−.04	.00	−.01	−.02	−.02	−.07
Executive Function	WCST	16.82 (12.36)	−.05	−.05	−.03	−.02	.02	−.06	.02	−.02
	Stroop – CW	30.22 (10.06)	.04	−.01	−.08	.04	−.08	.06	−.10	−.11
Learning and Recall	HVLT learning	22.98 (4.11)	−.03	.01	−.09	.04	−.09	.01	−.10	−.11
	HVLT recall	7.85 (2.67)	−.05	.01	−.10	.08	−.11	.00	−.12*	−.12*
	BVMT learning	18.17 (7.88)	−.01	.01	−.12*	.04	−.13*	−.02	−.15**	−.15*
	BVMT recall	7.35 (3.28)	−.06	−.08	−.17**	−.03	−.18*	−.07	−.20**	−.20**
Language	COWAT	24.10 (9.16)	−.02	−.04	−.06	−.01	−.01	.04	−.02	−.07
	Animals	12.18 (3.08)	−.02	−.02	−.11	.07	−.09	.00	−.11	−.14*
	CTQ	46.49 (19.28)	−.21**	−.19**	−.15**	−.11	−.10	−.20**	-	-
	CES-D	11.96 (14.97)	−.44**	−.45**	−.37**	−.29**	−.34**	−.43**	−.31**	−.31**
	DTS	17.38 (30.41)	−.35**	−.38**	−.27**	−.20**	−.34**	−.31**	−.12**	−.24**

* *p* < .05, **p < .01; CTQ = Childhood Trauma Questionnaire, CES-D = Center for Epidemiologic Studies Depression Scale, DTS = Davidson Trauma Scale; WAIS-II DS = Wechsler Adult Intelligence Scale III, Digit Symbol; WAIS-III SS = Wechsler Adult Intelligence Scale, III Symbol Search; PASAT = Paced Auditory Serial Addition Test; WMS-III SS = Wechsler Memory Scale III, Spatial Span; WCST = Wisconsin Card Sort Task, number of perseverative responses; Stroop CW = Stroop Color Word Test; Hopkins Verbal Learning Test – revised (HVLT-R); BVMT-R = Brief Visuospatial Memory Test – revised; COWAT = Controlled Oral Word Association Test, number correct.

Finally, we examined the associations between occupational and social resilience and neurocognitive functioning metrics found to be significant in the previous step by entering age, education, HIV status, depressive symptoms, and PTSD symptoms in step one of a hierarchical regression model given the known effect of these variables on neurocognitive performance (Devlin et al., 2012; McClintock et al., 2010; Scott et al., 2015), and then entering the neuropsychological variable in the second step. Regression coefficients for the second step of each model, as well as the *R^2^* change, are reported in Table 3. Only non-verbal memory (BVMT recall) contributed unique variance (*ΔR^2^* = .013, *ΔF* (6, 287) = 12.50 *p *= .033) to the association with greater *social* resilience, *B* = −0.04, *SE* = .019, *p *= .033. All other domains of neurocognitive functioning were non-significant for an effect on *social* resilience. Non-verbal memory (BMVT recall) also contributed unique variance (*ΔR^2^* = .014, *ΔF* (6, 288) = 9.48 *p *= .026) to the association with greater *occupational* resilience, *B* = -.04, *SE* = .019, *p *= .026. Also, semantic verbal language fluency (Animals) contributed unique variance to (*ΔR^2^* = .014, *ΔF* (6, 288) = 9.46, *p *= .027) to the association with *occupational* resilience, *B* = −0.04, *SE* = .018, *p *= .027. Finally, processing speed (WAIS III – Symbol Search) contributed unique variance to (*ΔR^2^* = .012, *ΔF* (6, 288) = 9.33, *p *= .04) to the association with *occupational* resilience, *B* = −0.02, *SE* = .008, *p *= .04. All other associations between neurocognitive domains and *occupational* resilience were not significant.

**Table 3.** Second step coefficients for final multiple hierarchical regression models isolating the contribution of neurocognitive variables to predicting social and occupational resilience above and beyond the effects of age, education, HIV status, and depressive and PTSD symptoms.

	Social Resiliency				Occupational Resiliency			
Variables	*B*	*SE (B)*	*Β*	*Δr^2^*	*B*	*SE (B)*	*β*	*Δr^2^*
Model 1								
Age	.02	.01	.14		.02	.01	.17**	
Education	−.10	.04	−.15*		−.02	.04	−.03	
HIV status	−.14	.12	−.07		−.09	.11	−.05	
CES-D	−.02	.00	−.24***		−.02	.00	−.27***	
DTS	−.01	.00	−.21**		−.00	.00	−.08	
**WAIS-III DS**	−.00	.01	−.06	.002	−.01	.01	−.14	.010
Model 2								
Age	.02	.01	.15*		.02	.01	.19***	
Education	−.10	.04	−.15*		−.02	.04	−.04	
HIV status	−.14	.12	−.07		−.08	.11	−.04	
CES-D	−.02	.00	−.24***		−.02	.00	−.28***	
DTS	−.01	.00	−.21**		−.00	.00	−.09	
**WAIS-III SS**	−.01	.01	−.06	.003	−.02	.01	−.13*	.012*
Model 3								
Age	.02	.01	.16**		.03	.01	.22***	
Education	−.11	.04	−.17		−.05	.04	−.08	
HIV status	−.14	.12	−.07		−.08	.12	−.04	
CES-D	−.02	.00	−.24***		−.02	.00	−.27***	
DTS	−.01	.00	−.21**		.00	.00	−.08	
**HVLT learning**	−.01	.01	−.05	.002	−.01	.01	−.06	.003
Model 4								
Age	−.02	.01	.16**		.03	.01	.21***	
Education	−.10	.04	−.16**		−.04	.04	−.07	
HIV status	−.14	.12	−.07		−.08	.11	−.04	
CES-D	−.02	.00	−.23***		−.02	.00	−.26***	
DTS	−.01	.00	−.22***		−.00	.00	−.09	
**HVLT delayed recall**	−.03	.02	−.08	.006	−.03	.02	−.08	.006
Model 5								
Age	.02	.01	.15*		.03	.01	.20**	
Education	−.10	.04	−.16**		−.04	.04	−.07	
HIV status	−.15	.12	−.07		−.09	.12	−.04	
CES-D	−.02	.00	−.24***		−.02	.00	−.28***	
DTS	−.01	.00	−.21**		.00	.00	−.07	
**BVMT learning**	−.01	.01	−.07	.004	−.01	.01	−.08	.004
Model 6								
Age	.02	.01	.13*		.02	.01	.19**	
Education	−.09	.04	−.14*		−.03	.04	−.05	
HIV status	−.17	.12	−.08		−.11	.12	−.06	
CES-D	−.02	.00	−.23***		−.02	.00	−.27***	
DTS	−.01	.00	−.20**		.00	.00	−.07	
**BVMT delayed recall**	−.04	.02	−.13*	.013*	−.04	.02	−.14*	.014*
Model 7								
Age	.02	.01	.16**		.03	.01	.21***	
Education	−.10	.04	−.16**		−.04	.04	−.06	
HIV status	−.15	.12	−.08		−.10	.12	−.05	
CES-D	−.02	.00	−.24***		−.02	.00	−.28***	
DTS	−.01	.00	−.21**		−.00	.02	−.08	
**Animals**	−.03	.02	−.09	.006	−.04	.02	.13*	.014*

* *p* < .05, **p < .01, ***p < .00; CES-D = Center for Epidemiologic Studies Depression Scale; DTS = Davidson Trauma Scale

## 4. Discussion

The primary aim of the present study was to identify neurocognitive correlates of social and occupational resilience among black South African women with a history of exposure to childhood trauma. Social and occupational resilience were targeted, given the importance of these functions in the under-resourced settings in which the women in our sample function in everyday life. Overall, our study findings offered mixed support for our hypotheses. While we did note significant associations between social and occupational resilience in specific domains of neurocognitive functioning, primary results were in the *opposite* direction than we hypothesized. At a univariate level, non-verbal learning (BMVT learning) and memory (BMVT recall), as well as verbal memory (HVLT recall) and processing speed (WAIS-III Digit-Symbol), were *inversely* associated with social and occupational resilience, while semantic fluency (Animals) was *inversely* associated with occupational resilience. In our hierarchical multivariate regression models, depressive symptoms and trauma symptoms were inversely associated with resilience, as expected. Also, older age was associated with increased resilience, consistent with prior studies demonstrating an association between greater resilience and increasing age (Hamarat, Thompson, Steele, Matheny, & Simons, 2002). However, while semantic verbal fluency (Animals), processing speed (WAIS-III Symbol Search), and non-verbal memory (BMVT delayed recall) continued to predict occupational resilience when partialling out the effects of age, education, HIV status, depressive and posttraumatic stress symptoms, the direction of these effects were in the opposite direction than what we hypothesized, such that *lower* neurocognitive scores were associated with greater resilience. A similar pattern was found for non-verbal memory (BMVT delayed recall) and social resilience. As depressive and trauma symptoms were generally negative predictors of resilience, therefore supporting the validity of our measure of resilience, we proceed to interpret our findings considering our primary aim and the three methodological approaches we adopted that had not been used in prior work.

The first unique methodological approach taken in this study to address limitations in the resilience literature introduced by assessing resilience more generally without consideration of the specific type of traumatic exposure, in this case childhood abuse, was to define resilience using our regression-based approach. This is important, because different types of traumatic exposure have differential effects on outcome (Brewin, Andrews, & Valentine, 2000). Trauma occurring in early life may have particularly strong effects on neurocognitive function given the multiple developmental changes in the brain that are occurring and the subsequent effects on extinction learning (Caspi et al., 2003; Gould et al., 2012; Pattwell et al., 2012; Slopen, Koenen, & Kubzansky, 2014). Animal studies have shown that early life stress permanently affects extinction learning and fear-related memory (Chocyk et al., 2014). Though highly speculative, it could be that deficits in fear related learning and memory could fail to inhibit avoidance behaviours because learning and consolidation of the feared event is inhibited. This could have adaptive implications when it comes to attempting to carry out daily activities in a risky environment because individuals might be more willing to risk exposure to stimuli in the absence of a feared memory (see Teicher, Samson, Anderson, & Ohashi, 2016; Vythilingam et al., 2002). Subsequent increased exposure would then provide opportunities for positive experiences, increased reward, and mastery. Alternatively, deficits in fear memory learning may threaten adaptive functioning because failure to associate learned cues with threat can increase risk for future exposure. These alternative explanations can only be reconciled with longitudinal or experimental studies, and future research should consider assessing neuropsychological functioning prior to exposure to fully explore causality.

A second methodological approach unique to the present study was to operationalize resilience in a way that affords the flexibility to assess functioning across culturally diverse contexts. This approach is advantaged over prior studies (Stein, Campbell-Sills, & Gelernter, 2009; Wingo et al., 2010) insofar as it reflects a much-needed conceptual emphasis on the importance of contextually appropriate measures of resilience, which in turn offers greater specificity regarding those domains of preservation that might be particularly relevant to the South African setting. The ability to deploy successful adaptive strategies in stressful contexts depends on the demands of the situation as well as the priorities of the individual deploying those strategies (Bonanno & Burton, 2013; Hayman et al., 2016), and our results regarding negative associations between some neurocognitive domains and improved functioning suggest an important, but counter intuitive, person-environment fit in the deployment of adaptive strategies.

Our third unique methodological approach of using a performance-based measurement approach to estimate neurocognitive outcomes that are potentially associated with resilience departs from prior work that relies solely on self-report assessments of resilience (e.g. Greeff & Loubser, 2008; Phasha, 2009). Taking this approach suggested possible mechanisms of action that might promote resilience after exposure to trauma. For example, though highly speculative, our findings regarding associations among non-verbal memory (BMVT recall) and social and occupational resilience could be consistent with models of post-exposure response to traumatic stimuli that feature components of fear memory and modulation as mechanisms in both recovery and pathological sequelae (Gershman & Hartley, 2015; Parsons & Ressler, 2013; Wingo et al., 2010). These models suggest that the maintenance of PTSD after fear exposure, as well as extinction of fear memories, occurs in three primary regions within the limbic system including the prefrontal cortex, the amygdala, and the hippocampus, known to be correlated with tests of non-verbal memory (Koenen et al., 2001; Mahan & Ressler, 2012). Though highly speculative, it may be that lower non-verbal memory reduces fear memory consolidation, thereby reducing vulnerability to developing PTSD and associated functional impairments (e.g. occupational functioning). However, this explanation is highly speculative, and the cross-sectional nature of our study limits the extent to which any such conclusion could be made.

Because functional status was assessed using a self-report inventory, we cannot rule out that our findings are an artefact of bias introduced by this method of evaluation. Higher self-reported occupational functional status could be an artefact of neurocognitive deficits in semantic fluency (e.g. Animals) and processing speed (e.g. WAIS-III SS) because deficits in these domains may result in perceived higher functional status because these very deficits attenuate the ability to accurately evaluate functional well-being. That is, individuals may therefore perceive they are doing better than they are. Similar patterns of differences between perceived functioning and performance-based assessment in executive functioning domains has been reported elsewhere (Buchanan, 2016).

Several limitations should be taken into consideration when interpreting the results of this study. Most importantly, the cross-sectional nature of the study makes it impossible to determine whether the relationships we observed were risk factors, mediators, or outcomes. For example, it could be that women who had higher occupational functioning developed better non-verbal memory capacity, thereby explaining the association. Second, given that resilience is best defined as a response to a marker event, the lack of prospective data is a limitation (Bonanno, 2012). Third, we relied on self-reported assessment of functional status, thereby introducing considerable bias. Future studies should employ observational measures of functional status.

Furthermore, our measure of childhood trauma did not incorporate an assessment of the interval between exposure to childhood trauma and adult neurocognitive functioning, nor did it measure age of exposure, chronicity, polyvictimization, or current treatment status. We attempted to control for some exogenous variables that could influence the association between neurocognitive functioning and resilience such as age, education, and HIV status, but future prospective studies are needed. A further limitation to the present study is the potentially inflated Type 1 error rate potentially introduced by testing models with each neuropsychological measure considered separately. While several of the neuropsychological tests considered in this study are designed to assess independent elements of neurocognitive functioning, they tend to be correlated and the potential of non-independence between neuropsychological test subscale scores suggested independent entry in hierarchical regression models was warranted.

The negative health consequences of exposure to adversities such as racism (Williams, 1999), interpersonal violence (Dutton et al., 2006), and childhood abuse (Springer et al., 2007) include higher rates of mood and anxiety disorders (Kessler, Davis, & Kendler, 1997) as well as impaired social and occupational functioning (Amaya-Jackson et al., 1999). Further investigation into potential pathways to resilience that could ameliorate the negative health consequences of adversity exposure is therefore indicated (see Wingo et al., 2017). These study findings suggest that there may be unexpected pathways to resilience after trauma, and further research should consider assessing neurocognitive domains and functional status using methods described here, to determine if the inverse associations found in this study are extended to other populations. Identification of neurocognitive domains associated with functional resilience could suggest important new avenues for both prevention and treatment of post-exposure psychopathology.
